# Investigation of temporomandibular dysfunction in patients with breast cancer–related lymphedema

**DOI:** 10.1007/s00520-025-09500-y

**Published:** 2025-05-07

**Authors:** Arife Akbulut Bayrak, Merve Yasemin Tekbudak, Serap Gultekin, Ilke Keser

**Affiliations:** 1 Department of Physiotherapy and Rehabilitation, Faculty of Health Sciences, Ankara Yildirim Beyazit University, Ankara, Turkey; 2 Department of Physiotherapy and Rehabilitation, Institute of Health Sciences, Gazi University, Ankara, Turkey; 3 Department of Statistics, Faculty of Science, Gazi University, Ankara, Turkey; 4 Department of Radiology, Faculty of Medicine, Gazi University, Ankara, Turkey; 5 Department of Physiotherapy and Rehabilitation, Faculty of Health Sciences, Gazi University, Ankara, Turkey

**Keywords:** Breast cancer, Lymphedema, Temporomandibular joint, Posture

## Abstract

**Purpose:**

This study aims to investigate temporomandibular joint dysfunction (TMD) in patients with breast cancer-related lymphedema (BCRL) and to determine the relationship between TMD severity and posture, range of motion (ROM), psychological status, and lymphedema (LE).

**Methods:**

Sociodemographic characteristics and previous treatments of 38 individuals included in the study were recorded. The Craniomandibular Dysfunction Index for Clinical (CMDIC) and visual analog scale (VAS) evaluated TMD symptoms, signs, and pain. Patients were divided into three groups: mild, moderate, and advanced TMD, according to CMDIC results. LE stages of the patients were recorded. The severity of LE, range of motion (ROM), anxiety and depression, and posture analysis were evaluated with circumference measurement, goniometer, Turkish version of the Hospital Anxiety and Depression Scale (HAD), photogrammetric assessment method, and inclinometer, respectively.

**Results:**

Different degrees of TMD were detected in all patients. There was a significant difference between the groups regarding post-op period duration, work status, posture, and ROM (*p* < 0.05). The groups’ LE stage, LE severity, and scores of HAD and VAS were similar (*p* > 0.05).

**Discussion:**

According to the results of this study, TMD is highly prevalent in BCRL patients. It is necessary to monitor TMD and its impact on the range of motion and posture. The post-op period duration can determine the development of TMD in this patient group.

**Trial registration:**

NCT06669910, date of registration: 01.11.2024, “retrospectively registered”.

## Introduction

Breast cancer, the most common type of cancer in women [1], is observed in 24.9% of women in Türkiye [2]. Interventions such as surgery, chemotherapy, radiotherapy, and hormone therapy applied in breast cancer treatment reduce the disease’s mortality rate and increase the morbidity rate. As a result of breast cancer surgeries, problems such as pain, upper limb lymphedema, and shoulder joint range of motion (ROM) restriction (especially flexion and external rotation) occur in patients, which reduce the quality of life of patients [3]. Scar tissue, asymmetry in muscle strength, fibrosis, and osteoporosis due to surgery, radiotherapy, and hormone therapy after cancer treatments can lead to postural disorders such as scapular asymmetry, increased thoracic kyphosis, and scoliosis [4–6]. In addition, pain, swelling, loss of strength, and limitation of upper extremity joint movements may occur in the affected arm due to lymphedema (LO), which is one of the common problems encountered in breast cancer patients [7]. As a result of pain and tension due to increased arm weight bearing, a posture with the shoulder elevated develops [8].

In addition to the postural changes caused by LE, psychological effects, decreased skin elasticity, and development of fibrosis due to radiation therapy may also cause contracture in the cervical muscles [8, 9]. According to the American Academy of Orofacial Pain, there is a relationship between the cervical region and temporomandibular joint dysfunction (TMD). Studies show a biomechanical relationship between the craniofacial region and the cervical spine [10]. In their studies on individuals with and without TMD, Yildiz et al. reported that neck flexion, extension, and lateral flexion joint ROM were less in individuals with TMD than in those without TMD [11]. In postural disorders where the head is anteriorly positioned, shoulders are protracted; cervical lordosis, thoracic kyphosis, and lumbar lordosis increase; the temporomandibular joints (TMJ) effect may be formed by changed posture of the mandible and spasm of masticatory muscles [12, 13]. TMD is a group of musculoskeletal problems that may be unilateral or bilateral, originating from any one or a combination of the temporomandibular joints (TMJ), masticatory muscles, and surrounding bone and soft tissues. Masticatory muscle pain, TMJ limitation, and head and neck pain are common symptoms of TMD [14].

It was hypothesized that postural disorders, neck and shoulder problems seen in breast cancer survivors may cause TMJ involvement in this patient group. Psychological effects are known to cause muscle pain by increasing muscle tone and TMD, along with changes of mandibular position [10, 15]. However, to the best of our knowledge, no study examining TMD in patients with breast cancer-related lymphedema (BRCL) was found in the literature. Given that TMD and breast cancer frequently occur in women of comparable ages, it was thought that the potential for TMD in patients diagnosed with breast cancer should be investigated [16, 17]. The aim of this study was to examine TMD in patients with BCRL. The secondary aim of the study was to investigate the effects of posture, neck and shoulder ROM, psychological status, and LE severity on TMD severity in BCRL patients.

## Material and method

This study was conducted with patients who applied to Gazi University Hospital between November 2019 and March 2020, who were diagnosed with breast cancer and completed the active treatment stages and were referred to receive recommendations at the Oncology Rehabilitation Unit of Gazi University Faculty of Health Sciences, Department of Physiotherapy and Rehabilitation.

After the patients were informed about the study, those who agreed to participate in the study were asked to sign the “Informed Consent Form.” Approval for this study was obtained at the meeting of the Clinical Research Ethics Committee of Gazi University Faculty of Medicine Clinical Research Ethics Committee on 25.11.2019 (Decision 200).

### Individuals

Female patients aged 18–75 years who were treated for unilateral breast cancer and developed breast cancer–related LE were included in the study. Patients with general joint diseases affecting the head and neck region that may cause TMD (e.g., rheumatoid arthritis), jaw fracture, trauma or orthognathic surgery history, cervical disc herniation, congenital disease, and facial paralysis; patients who have been treated for TMD for the last 3 months; and patients who use antidepressant drugs were excluded.

### Evaluation methods

All patients were evaluated by the same physiotherapist. Data on age, height, body weight, dominant side, side affected by breast cancer, postoperative period duration, and treatment modalities (surgery/chemotherapy/radiotherapy/hormone therapy) for breast cancer were recorded. Body mass index (BMI) was calculated [18].

LE stages were determined according to the criteria set by the International Lymphology Association [19]. To determine the severity of LE, circumference was measured at 4-cm intervals from both arms. The difference between the two limbs was classified as “normal” if it was up to 1.5 cm, “mild” if it was between 1.5 and 3 cm, “oderately severe” if it was 3–5 cm, and “severe” if it was over 5 cm [20].

*The Clinical Craniomandibular Dysfunction Index (CCMDI)* was used to evaluate TMD symptoms and signs in detail. The CCMDI consists of five components: restricted mandibular normal joint movement (NJM), pain during mandibular NJM, pain during TMJ palpation, pain during masticatory muscle palpation, and pain during mandibular function. For each component, 3 points can be given as 0, 1, or 5 points, and according to the scores, individuals are rated as non-TMD (0 points), mild TMD (1–4 points), moderate TMD (5–9 points), and severe TMD (10–25 points) [21].

*The intensity of TMJ pain* was assessed by the visual analog scale (VAS) at rest and during activity [22]. Patients were asked to mark the point that best describes their pain on a 10 cm line (0, no pain at all; 10, intolerable pain). Pain intensity with activity was evaluated during mouth opening, mouth closing, and chewing gum for 60 s. Patients were asked to chew gum using both sides for 60 s. At the end of this period, the patients evaluated their pain caused by TMJ, according to the VAS [23].

*For posture analysis*, the “Posture Analysis Method with Photography,” reported to be the most accurate and objective method in the literature, was used [24]. This method was applied according to the procedure followed by Pausic et al. [25]. A poster consisting of 5 × 5 squares, 85 cm wide and 2 m long, was fixed to the wall starting from the floor. The camera was placed 1.5 m away from the patients and 115 cm above the ground using a tripod. Each patient was photographed from the right side, from the left side, from the front, and the back so that reference points (eye canthus, tragus, earlobe, cervical (C)7 spinous protrusion, acromion, midpoint of tubercle major with posterior aspect of acromion, lowest point of scapula, trochanter major, spina iliaca anterior superior (SIAS), and spina iliaca posterior superior (SIPS) were visible from an equal distance. Craniohorizontal, craniovertebral, sagittal shoulder posture, and the angle formed by the anterior posture of the trunk and head were measured with the verifications drawn from reference points using the ImageJ program. The symmetry of the scapula, acromion, SIPS, and SIAS was evaluated [24, 26].

*The kyphosis and lordosis angles* of the patients were measured with a Baseline® Digital inclinometer while the patients were standing in a comfortable position. The measurement of the angles between the spinous processes of the vertebrae was performed with an inclinometer from the degree of thoracic kyphosis, thoracal (T) 1–2 and T12–lumbal (L)−1. To determine the degree of lumbar lordosis, T12-L1, and Sacral (S) 2–3, the angle between the spinous decussations of their vertebrae was measured, and the determined degrees were summed. The reference values for thoracic kyphosis are 20–45, while for lumbar lordosis are 20–40 [27, 28].

*The ROM measurement of cervical and shoulder joints* was performed with a Baseline® universal goniometer with a plastic 360 dial. Flexion, extension, right-left lateral flexion, and rotation angles were measured for the cervical region, and flexion, abduction, and external and internal rotation ROM angles for the shoulder joint were measured. Measurements were performed in the sitting position for the cervical region and the supine position for the shoulder. Each measurement was repeated 3 times, and the mean values were recorded [29].

The Turkish version of the Hospital Anxiety and Depression Scale (HAD) was used to determine *anxiety and depression*. The HAD is a 4-Decker Likert-type scale with a score between 0 and 21, consisting of a total of 14 questions, 7 of which assess anxiety and 7 of which assess depression [30].

### Statistical analysis

The data were analyzed using IBM SPSS Statistics (Version 25) [31]. Descriptive statistics were given for the continuous variables in the study, and frequency values and percentile values were given for the discrete (sortable/classifiable) variables. Before parametric statistical analyses were performed, the Shapiro–Wilk test was used to check whether the continuous variables were suitable for normal distribution. One-way analysis of variance (one-way ANOVA) was applied for the mean comparison of variables suitable for the normal distribution. The Kruskal–Wallis *H* test, the nonparametric equivalent of ANOVA, and the Mann–Whitney *U* test, the nonparametric equivalent of Student’s *t*-test, were used for mean comparisons of variables that did not conform to the normal distribution. In addition, Spearman correlation coefficients were calculated to examine the pairwise correlation values of the variables in the study. All statistical analyses were conducted with a minimum 5% margin of error.

## Results

**Fig. 1:**
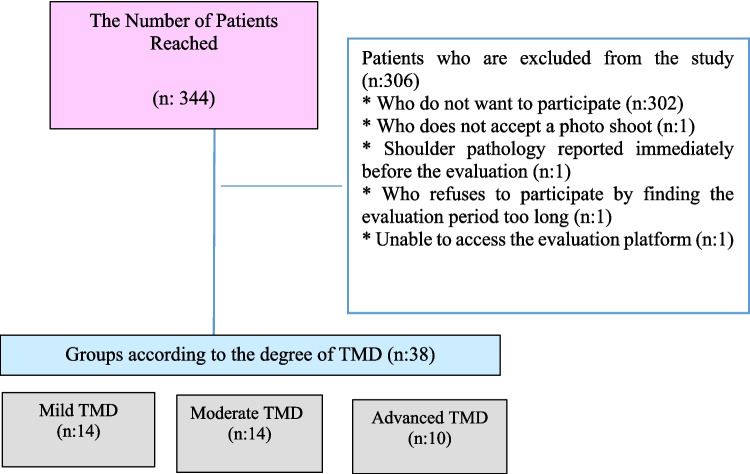
Flow chart of the study

Of the 344 patients informed about the study, 42 agreed to participate. The reasons for 306 patients being excluded from the study are stated in the following flow chart. TMD was detected according to CCMDI in all 38 patients, in whom all evaluations were completed. Patients were examined in three groups according to their degree of TMD: mild TMD (*n* 14), moderate TMD (*n* 14), and advanced TMD (*n* 10) (Fig. 1).

When the three groups were compared, it was seen that the degree of TMD also progressed as the age and BMI values of the patients increased. However, this increase was not significant (*p* > 0.05). When the sociodemographic characteristics and resumes of the groups were examined, it was seen that the employment rate of patients in the advanced TMD group was less than that of the other groups (*p* 0.040). It was found that an increase in the postoperative period duration also increased the degree of TMD (*p* 0.001). There was no significant difference between the groups regarding the dominant arm, affected arm, and treatment methods applied (*p* > 0.05). When the jaw pain status was examined, the pain in the right jaw was found to be different in mouth opening(*p* 0.043). This difference was between the advanced group and the moderate (*p* 0.048) and mild (*p* 0.048) groups. Other parameters were found to be similar (*p* > 0.05) (Table 1).

**Table 1:** Characteristics of patients according to TMD degrees and information about their previous treatment

		**TMD degree**					
		**Mild **(*n* 14)		**Moderate **(*n* 14)	**Advanced **(*n* 10)		
		**Mean ± SD**				***F***	***p***
Age (years)		49±10	52±9		58±9	2.892	0.069^a^
BMI (kg/m^2^)		28.23±5.15	27.24±5.91		30.05±5.31	0.769	0.471^a^
		***n***	***N***		***n***	***χ***^2^	***p***
Working status	Working	8	7		4	2.220	**0.040**^b^
	Retired	4	4		2		
	Never worked	2	3		4		
Dominant arm	Right	13	14		10	1.760	0.360^b^
	Left	1	0		0		
The affected arm	Right	10	4		6	5.450	0.120^b^
	Left	4	10		4		
Surgical	BPS	7	5		3	4.130	0.070^b^
	MRM	6	4		5		
	Mastectomy	1	5		2		
Chemotherapy	Yes	10	11		9	1.160	0.540^b^
	No	4	3		1		
Radiotherapy	Yes	10	8		8	1.500	0.160^b^
	No	4	6		2		
Application of radiotherapy to the axilla	Yes	8	5		8	4.650	0.100^b^
	No	6	9		2		
Application of radiation therapy to the breast	Yes	10	8		8	1.500	0.160^b^
	No	4	6		2		
Application of radiotherapy to the supraclavicular region	Yes	1	3		1	1.360	0.220^b^
	No	13	11		9		
Hormone therapy	Yes	12	11		9	0.610	0.210^b^
	No	2	3		1		
		**Median (IQR)**					***p***
Surgical duration (year)		1 (1–2)	4.5 (3–8)		7.5 (2–10)		**0.001**^c^
VAS resting right		0 (0–0)	0 (0–0)		0 (0–0)		0.532^c^
VAS rest left		0 (0–0)	0 (0–0)		0 (0–0.50)		0.284^c^
VAS mouth opening right		0 (0–0)	0 (0–0)		0 (0–2.90)		**0.043**^c^
VAS mouth opening left		0 (0–0)	0 (0–0.40)		0 (0–0.80)		0.089^c^
VAS mouth closure right		0 (0–0)	0 (0–0)		0 (0–0)		1.000^c^
VAS left mouth closure		0 (0–0)	0 (0–0)		0 (0–0)		0.209^c^
VAS chewing right		0 (0–0)	0 (0–0.50)		0 (0–2)		0.450^c^
VAS chewing left		0 (0–0)	0 (0–2.10)		0 (0–1.60)		0.309^c^

*n* number of people, *BPS* breast protective surgery, *MRM* modified radical mastectomy, *VAS* visual analog scale, *BMI* body mass index, *SD* standard deviation, *Min* minimum value, *Max* maximum value, ^a^analysis of variance-ANOVA, *F* ANOVA test statistics, ^b^chi-square test, χ^2^ chi-square test statistics, ^c^Kruskal-Wallis *H* test, *IQR* interquartile range

Bold entries indicate:* p* < 0.05

When the posture analyses were evaluated, it was found that there was a significant difference in the degrees of kyphosis between the groups(*p* 0.020). In pairwise comparisons, it was found that the difference in kyphosis was due to mild TMD and moderate TMD groups (*p* 0.006). No significant difference was found when other parameters were compared (*p* > 0.05) (Table 2).

**Table 2:** The relationship between posture and temporomandibular joint dysfunction

		**TMD degree**					
		**Mild **(*n *14)	**Moderate **(*n* 14)		**Advanced **(*n* 10)		
		***n***	***N***		***n***	***χ***^2^	***p***
Scapular lower point	Equal	6	4	4		1.610	0.860^a^
	Above right	1	3	1			
	Above left	7	7	5			
Acromion	Equal	5	5	2		8.880	0.100^a^
	Above right	6	2	7			
	Above left	3	7	1			
SIAS	Equal	5	3	2		8.810	0.840^a^
	Above right	6	2	6			
	Above left	3	9	2			
SIPS	Equal	9	10	8		4.000	0.110^a^
	Above right	4	2	0			
	Above left	1	2	2			
		**Mean±SD**				***F***	***p***
Kyphosis		36.66±8.06	47.46±8.52	43.12±12.94		4.375	**0.020**^b^
Lordosis		38.77±6.94	37.72±8.45	32.04±12.59		1.710	0.196^b^
Body angle		9.79 ±1.75	9.93±2.99	9.80±3.44		0.11	0.989^b^
Craniohorizontal angle		19.65±5.42	19.61±6.18	15.21±4.25		2.418	0.104^b^
Craniovertebral angle		54.50±4.81	55.14±7.70	50.39±7.22		1.672	0.203^b^
		**Median (IQR)**					***p***
Sagittal shoulder posture angle		13.23 (7.37–16.50)	22.77 (16.24–26.46)	13.01 (11.57–26.92)			0.056^c^
Anterior posture angle of the head		178.50 (176.82–180)	178.27 (177.10–180)	178.07 (175.44–180)			0.905^c^

*TMD* temporomandibular joint dysfunction, *SD* standard deviation, *Min* minimum value, *max* maximum value, *n* number of people, SIAS Spina Iliaca Anterior Superior, *SIPS* spina iliaca posterior superior, *χ*^2^ chi-square test statistic, *F* ANOVA test statistic, *IQR* interquartile range. ^a^Ki-kare testi. ^b^ANOVA. *F*, ANOVA test istatistiği. ^c^Kruskal-Wallis *H* test

Bold entries indicate: *p *< 0.05

The ROM values of the groups were compared; the right lateral neck flexion (*p* 0.021) and left shoulder internal rotation (*p* 0.031) ROM values were significantly different. It was determined that the difference in right lateral flexion of the neck was caused by the higher ROM value of the mild TMD group compared to the moderate TMD (*p* 0.03) and advanced TMD (*p* 0.01) groups. Other values in the neck and shoulder were not different between the groups. Anxiety and depression scores according to the HAD were also found to be similar between the groups (*p* > 0.05) (Table 3).

**Table 3:** Normal joint movement and anxiety-depression according to temporomandibular joint dysfunction

	**TMD degree**				
	**Mild **(*n* 14)	**Moderate **(*n *14)	**Advanced **(*n* 10)		
	**Mean ± SD (Min–Max)**	**Mean ± SD (Min–Max)**	**Mean ± SD (Min–Max)**	***F***	***p***
Neck flexion	43.21 ± 10.11 (25–65)	44.35±5.69 (35–55)	39.8±8.18 (25–50)	0.938	0.401^a^
Neck extension	32±9.27 (15–50)	34.35±8.75 (20–55)	32.5±6.34 (20–40)	0.299	0.744^a^
Neck rotation to the right	51.21±5.65 (40–62)	48.78±5.80 (40–55)	46±9.66 (30–65)	1.644	0.208^a^
Neck left rotation	49.21±8.77 (32–65)	45.71±8.50 (30–55)	43.8±6.82 (35–60)	1.369	0.268^a^
Lateral flexion of the neck to the right	45.43±9.96 (28–60)	39.43±4.80 (30–45)	37.20±6.01 (25–45)	4.145	**0.024**^a^
Right shoulder external rotation	71.42 ±2.43 (50–90)	72.71±13.15 (40–90)	68.6±15.99 (40–90)	0.268	0.767^a^
Anxiety	5.78±2.57 (1–10)	7.42±3.47 (1–14)	6±3.05 (1–10)	1.154	0.327^a^
Depression	4.35 2.46 (1–8)	4.71 3.29 (0–10)	5.20±3.29 (1–10)	0.228	0.797^a^
	**Median IQR**	**Median IQR**	**Median IQR**		***p***
Left lateral flexion of the neck	41 (30–48)	35 (30–40)	32.50 (30–40)		0.216^b^
Right shoulder flexion	176 (160–180)	180 (170–180)	175 (170–180)		0.667^b^
Right shoulder abduction	161 (120–180)	180 (160–180)	170 (160–180)		0.312^b^
Right shoulder internal rotation	85 (80–90)	80 (80–85)	80 (60–85)		0.104^b^
Left shoulder flexion	180 (174–180)	172.5 (165–180)	170 (165–180)		0.385^b^
Left shoulder abduction	180 (170–180)	170 (132–180)	180 (145–180)		0.730^b^
Left shoulder internal rotation	85 (80–90)	80 (60–85)	77.5 (70–83)		**0.031**^b^
Left shoulder external rotation	82.5 (75–85)	80 (60–85)	70 (45–80)		0.062^b^

*TMD* temporomandibular joint dysfunction, *Min* minimum value, *Max* maximum value. ^a^ANOVA, ^b^Kruskal-Wallis *H*. *F*, ANOVA test statistic

Bold entries indicate:* p *< 0.05

It was observed that the degree of TMD increased as the stage and severity of LE increased, and no significant difference was found between the groups (*p* > 0.05) (Table 4).

**Table 4:** Association of lymphedema with temporomandibular joint dysfunction

		TMD degree			*χ*^2^	*p*
		Mild (*n* 14)	Moderate (*n* 14)	Advanced (*n* 10)		
LE stage	Stage 0	6	7	1	5.239	0.277
	Stage 1	5	3	4		
	Stage 2	3	4	5		
LE severity	Normal	8	7	2	5.157	0.552
	Light	3	4	4		
	Middle	2	1	1		
	Severe	1	2	3		

*TMD* temporomandibular joint dysfunction, *LÖ* lymphedema, *n* number of people superior (chi-square test), *χ*^2^ chi-square test statistic

Bold entries indicate: *p *< 0.05

## Discussion

This study is the first known study to examine TMD in BCRL patients, so the risk factors that may cause TMD have been investigated in detail. TMD involvement has been commonly identified in patients with BCRL. It has been determined that the post-op period duration, age, BMI, pain, postural changes (kyphosis), and neck and shoulder ROM limitations may differ according to the severity of TMD in BCRL patients with different TMD severities.

### Sociodemographic characteristics

While the age range in which breast cancer is frequently seen in studies is 40–49 years [32], the fact that the patients who participated in our study were in the 32–73 age range and had an average age of 52.55 years was attributed to the fact that a certain period of time passed until the patients applied to physiotherapy applications and were lost. Physiotherapy and rehabilitation applications are generally considered after the completion of breast cancer treatment. However, it is known that the cancer rehabilitation process should start when the individual is healthy before diagnosis and continue after the diagnosis period, specifically for breast cancer [33]. The fact that the degree of TMD progresses with increasing age and post-op period duration suggests that TMD may become a more serious problem in the chronic period in patients with BCRL. The reason for this was thought to be the increase in postural abnormalities in the patient as the duration of surgery increased, limitation of ROM due to disuse, and prolonged exposure to anxiety and depression [34]. The fact that the advanced TMD group had the highest BMI value may be due to increasing body weight and an increase in the weight difference and asymmetry between the affected and healthy arm [35].

Since the dominant and the affected sides showed homogeneous distribution between the groups, it was thought that it would not affect the results. The reason why the number of working patients was higher in the mild TMD group may be that the physical activity levels of working patients were higher than those of non-working patients [36]. In addition, the fact that working women have more socialization opportunities may have decreased the rate of psychological effects related to BCRL and thus the incidence of TMD.

The fact that radiation therapy was applied more to the breast in the mild TMD group, to the supraclavicular region in the moderate TMD group, and to the axilla in the advanced TMD group showed that the severity of TMD may be affected depending on the presence and degree of radiogenic fibrosis in the radiation therapy area. Multiple changes in many factors, such as antalgic posture, circulation, posture, and joint range of motion after radiation therapy, may be factors affecting the development and progression of TMD [37]. These associations may be more clearly determined in future studies examining the relationship between radiotherapy and TMD in detail. Chemotherapy and hormone therapy showed similar distributions between the groups. The effects of systemic and multifaceted side effects of these therapies on TMD may be examined in more detail in future studies.

### Lymphedema and temporomandibular joint dysfunction symptoms

In our study, 34.2%, 34.2%, and 26.31% of the 38 patients diagnosed with BCRL were found to have mild, moderate, and severe TMD, respectively. Since this is the first study in the literature on this subject in the BCRL patient group, a contribution to the literature has been made by obtaining basic information. Although the results between the LE stage and severity and TMD are not significant, it is observed that the degree of TMD progresses as the LE stage and severity increase. In BCRL patients, ROM limitations, postural changes, and psychological influences due to LE may cause TMD and increase the degree of TMD.

Among the TMD groups, the pain during mouth opening was observed most in the advanced TMD group. It has been shown that as the degree of TMD increases, pain increases, and patients’ quality of life and daily living functions may be affected.

### Range of motion

The reasons for the limitation of shoulder abduction, flexion, and external rotation ROM in the majority of patients are the restriction of upper extremity movements due to the weight and edema of the extremity with LE; the scar tissue formed as a result of the surgical incision applied to the axillary region, preventing the shoulder movements from being completed, breast surgeries, and radiotherapy. It has been thought that damage to the pectoral muscles as a result, a decrease in the elasticity of the tissues due to radiogenic fibrosis resulting from radiotherapy, and adhesion and shortening in the muscles may be effective [38]. Limitations in shoulder and neck ROM and increased asymmetry in the shoulder girdle are associated with TMD.

Studies have reported that fibrosis occurs in the muscles due to radiation therapy methods used in breast cancer patients and causes limitations in shoulder ROM [38]. In our study, left shoulder internal rotation differed between the groups, and there was a limitation in other shoulder ROM values in many patients. Increasing internal rotation limitation in BCRL patients increased the degree of TMD. This may be related to less use of internal rotation in activities of daily living and antalgic posture. The postural change may affect TMD.

Subasi et al. reported a relationship between neck ROM values and TMD [39]. It has been reported that neck flexion and extension movements contribute biomechanically to mouth opening and closing movements [40]. In this study, it is observed that the right lateral flexion of the neck decreases as the degree of TMD increases. Although the results are not significant, it is observed that an increase in the limitation of neck movements, such as flexions and rotations, causes progression in the degree of TMD. It is thought that TMD may develop due to limitation in shoulder and neck movements in patients with BCRL due to the side effects of the LE and the side effects of the treatment.

### Postural disorders

Studies show that postural disorders in which the head is forward, thoracic kyphosis, and lumbar lordosis increase the degree of TMD more [10]. Our study found a significant relationship between the increasing angle of kyphosis and the degree of TMD in patients. An increasing upper limb weight due to LE pulls the trunk anteriorly and increases the degree of kyphosis, which may be one of the potential factors that will cause an increase in the degree of TMD. Although lordosis, body angle, and shoulder posture were similar between the groups, postural disorders were observed in many of the patients.

Park and Bae reported in their study that scoliosis may also lead to TMD because it causes muscle imbalance in the cervical and thoracic regions, consequently restricting TMJ movements and causing imbalance in the masticatory muscles [41]. The reason why there was no difference between the groups in terms of asymmetry was thought to be the prevalence of trunk asymmetry in all groups. Postural abnormalities may have caused TMD in BCRL patients, but more studies examining the effects on the degree of TMD are needed.

### Psychological factors

It has been reported that patients diagnosed with breast cancer may develop anxiety and depression due to reasons such as having been diagnosed with cancer, cancer treatments and their side effects, development of LE, and shoulder ROM limitation [42]. According to a study done by Bonjardim et al. on 217 individuals, assessing the anxiety and depression levels of individuals with HAD reported a relationship between TMD and anxiety score [43]. In this study, although there was no significant difference between the anxiety and depression scores according to the HAD scale in the TMD and LE groups, the determination that the mean depression score increased as the degree of TMD progressed was explained as the tension in the neck and jaw muscles increased as the depression level of the patients progressed, which may increase the degree of TMD.

When the HAD scores are taken into consideration, it is seen that the anxiety level is generally higher than the depression level. During interviews with patients, it was stated that many patients had concerns about the possibility of breast cancer recurrence and the progression of LE, and they did not want to think about these issues. Therefore, it is thought that patients may not have been able to give answers that reflect their own feelings to the questions in the scale because they wanted to believe that they had overcome the psychological effects of the disease. This supports the view that patients have concerns about their disease.In addition, the HAD scale was reported to be insufficient in evaluating TMD [44]. It was concluded that future studies need to include more patients and evaluate anxiety and depression in more detail.

## Conclusion

This study is the first to examine the relationship between BCRL and TMD. Different degrees of TMD were detected in all BCRL patients evaluated within the scope of this study. The fact that a significant rate of TMD is seen in this patient group shows that TMD should be added to evaluation and treatment in future studies and clinical practice. Age, post-op period duration, working status, BMI, pain, postural changes (kyphosis), and neck and shoulder ROM limitations differ according to the degree of TMD in patients with BCRL. As a result, the treatment methods used in the treatment of breast cancer, the limitations and postural disorders that develop due to LE, and the patient’s exposure to these factors for a longer period of time after surgery may cause TMD and advance the degree of TMD. This study, as the first study examining TMD in BCIL patients, contributed to the literature by determining that TMD is an important problem in this patient group. It is important to reach a larger number of patients and examine TMD in BCIL patients in more detail in future studies.

## Data Availability

“Data is provided within the manuscript or supplementary information files”.
